# Behavioural Thermoregulatory Tactics in Lacustrine Brook Charr, *Salvelinus fontinalis*

**DOI:** 10.1371/journal.pone.0018603

**Published:** 2011-04-07

**Authors:** Andrea Bertolo, Marc Pépino, Julie Adams, Pierre Magnan

**Affiliations:** Centre de recherche sur les interactions basins versants – écosystèmes aquatiques (RIVE), Université du Québec à Trois-Rivières, Trois-Rivières, Canada; Freie Universitaet Berlin, Germany

## Abstract

The need to vary body temperature to optimize physiological processes can lead to thermoregulatory behaviours, particularly in ectotherms. Despite some evidence of within-population phenotypic variation in thermal behaviour, the occurrence of alternative tactics of this behaviour is rarely explicitly considered when studying natural populations. The main objective of this study was to determine whether different thermal tactics exist among individuals of the same population. We studied the behavioural thermoregulation of 33 adult brook charr in a stratified lake using thermo-sensitive radio transmitters that measured hourly individual temperature over one month. The observed behavioural thermoregulatory patterns were consistent between years and suggest the existence of four tactics: two “warm” tactics with both crepuscular and finer periodicities, with or without a diel periodicity, and two “cool” tactics, with or without a diel periodicity. Telemetry data support the above findings by showing that the different tactics are associated with different patterns of diel horizontal movements. Taken together, our results show a clear spatio-temporal segregation of individuals displaying different tactics, suggesting a reduction of niche overlap. To our knowledge, this is the first study showing the presence of behavioural thermoregulatory tactics in a vertebrate.

## Introduction

Temperature is recognized as one of the most important environmental factors controlling the energetics of ectotherms, affecting, among other things, their growth, reproduction, and distribution [1], [2]. Given the pervasiveness of temperature effects on these organisms, thermoregulation is key to their survival [3]. Because they cannot rely on endogenous thermoregulatory mechanisms (e.g., thermogenesis) to control their body temperature, ectotherms mainly use behavioural thermoregulation—the active selection of a given temperature—to optimize their metabolic processes in heterogeneous thermal habitats [3]. Basking reptiles and fish seeking thermal refuges are examples of organisms relying on behavioural thermoregulation to improve their locomotor performance [4] or to avoid conditions close to their tolerance limits [5].

Since processes such as growth or digestion can differ in their thermal optima [6], [7], organisms face trade-offs in their behavioural thermoregulatory decisions. One solution to cope with the presence of multiple optima is to select an intermediate temperature that allows a compromise among competing physiological processes (e.g., [8]). Another solution is to temporally vary the selected temperature to maximize the rate of different processes occurring at different times. For example, some fish are known to select one temperature to feed and another one to digest in order to maximize the overall rates of consumption [9] or digestion and growth efficiency [10]. The need to vary body temperatures to optimize cyclic events (e.g., diurnal feeding and nocturnal digestion) could therefore lead to cyclic thermoregulatory behaviours.

It has long been accepted that, within a given species, there is only one “final thermal preferendum” (i.e., the temperature actively selected independently of the previous thermal experience; *sensu* [11]), suggesting that thermal behaviour should be similar among conspecifics. However, the general validity of the final thermal preferendum paradigm has been challenged by a large number of experimental studies showing that temperature selection can be influenced by factors such as acclimation [12], [13], sex [14], intra- and inter-specific interactions [15], [16], and feeding [10], [17], [18]. Polymorphism could also favour the appearance of different thermoregulatory behaviours within populations, as shown in polymorphic insects for colour patterns [19]. Despite experimental evidence of within-population phenotypic plasticity in ectotherm thermal behaviour, the occurrence of alternative thermoregulatory tactics is still rarely considered explicitly when studying natural populations [e.g., 8,20].

Brook charr (*Salvelinus fontinalis*) is a good model for studying the plasticity of thermoregulatory behaviour in ectotherms. Several studies have shown that this species exhibits trophic polymorphism in some Canadian Shield lakes, with a pelagic form feeding on zooplankton and a littoral form feeding on benthic organisms [21]–[23]. Such within-population variability in both prey and habitat use suggest that there is the potential for brook charr to develop alternative tactics of thermoregulatory behaviour. Although trophic polymorphism is relatively common in freshwater fishes [24], the consequences of this phenomenon on the thermoregulatory behaviour have not yet been explored.

The goal of this study was to determine if clear patterns of thermoregulatory behaviour occur at the within-population level in brook charr, which would suggest the existence of distinct tactics. More specifically, we predicted that individual temperature signals can be decomposed into two components: (i) one main signal of cyclic (diel) behavioural thermoregulation shared by all individuals, similar to what is observed in other salmonids that spend the night in warm surface waters and move toward relatively deep waters to cool down during the day (e.g., [9]), and (ii) specific signals (e.g., in terms of mean temperature or crepuscular behaviour) overlapping with the main one and characterizing different groups of individuals. We show here how these questions can be addressed by extending spatially explicit eigenvector-based methods to multiple time series.

## Materials and Methods

### Study lake

The study was carried out in the summers of 2003 and 2005 in Lake Ledoux, Mastigouche Reserve (46°38′ N, 73°15′ W), Québec, Canada. Lake Ledoux is a typical small oligotrophic temperate zone lake with respect to surface area (11.9 ha), mean depth (5.5 m), maximum depth (17.0 m), and general physicochemical characteristics [25]. Brook charr is the only fish species in the lake, and sport fishing is rigorously controlled by the Québec Government [25]. Lake Ledoux offers a highly heterogenous thermal habitat since it is vertically stratified during summer, with optimal conditions for thermoregulatory behaviour (ranging from 9 to 20°C for brook charr; [26]). The lake was closed to fishing for the two summers.

### Thermo-sensitive radio transmitter attachment

Fish were captured in June and July 2003 and 2005 with Alaska traps covering the littoral (<2 m depth) and deeper (>4 m depth) zones of the lakes. Adult individuals (total length >250 mm) were lightly anesthetized with tricaine methanesulphonate (MS-222) and equipped with 4 g thermo-sensitive radio transmitters (ATS-F1970 model, Advanced Telemetry System, Isanti, Minnesota, USA). Transmitters were attached externally under the dorsal fin and fixed with nylon monofilament threaded through muscular tissues at two points [27]. The transmitter was placed on one side of the fish while the filament and fixation knot on the other side were kept away from the fish by a small rubber plate [28]. A neoprene cushion was placed between the fish and the rubber plate to avoid lesions due to rubbing. The tagging process usually lasted less than 1 min. Fish were disinfected with china green (0.1 mg/L) and then kept in an enclosure (3 m ×4 m ×6 m depth) for four days. Only fish in apparently good shape and behaving normally were released in the lake.

### Radio-tracking

In 2005, individual fish were localized on average every two days during both day (09:00–15:00) and night (9 pm–3 am) using a radio-receiver (model R2000, ATS) equipped with a loop antenna. To reduce disturbance, localizations were done from a 6.5 m boat powered by an electric motor. For each fish, we recorded the geographic coordinates (latitude/longitude; North American Datum 1983) using a Global Positioning System (GPS; hand-held Garmin E-Trex) equipped with a wide area augmentation systems (WAAS). Since radio tracking was conducted only during the day in 2003, these data were not included in the analyses.

### Temperature data

In 2003, the temperature profile of the lake was measured on average every two days from the surface to 10 m of depth every 0.5 m with a YSI model 33 S-C-T temperature probe (±0.5°C). In 2005, thermal stratification was surveyed using 20 thermographs (iBcod, Alpha Mach Incorporation, Mont St-Hilaire, Canada) fixed every 0.5 m from the surface to 10 m in depth. Temperature data were recorded each hour throughout the whole study period. Thermographs in the upper 4 m were protected by white bored plastic tubes to prevent warming from solar radiance.

A radio receiver (model R2100, ATS, Isanti, Minnesota, USA) was installed on a raft anchored in the middle of the lake. Two loop antennas oriented perpendicularly and connected with the radio receiver were used to pick up radio transmitters. A data logger (ATS-Data Collection computer) connected to the radio receiver recorded the pulse frequency of each transmitter every 30 min. The temperature was determined by a specific relationship (linear regression) between pulse frequency and temperature, which was calibrated for each transmitter. Mean hourly temperatures calculated for each transmitter were used in all analyses. Only transmitters whose spatial coordinates varied over time during a 30 d survey period were used in the analyses (Table 1). This allowed us to discard dead individuals (e.g., by predation or post surgical infection) as well as those who had lost their transmitters. This resulted in 33 individuals (17 in 2003 and 16 in 2005) being retained for the analyses. All analyses were done on one month of data, spanning from mid-June to mid-July in 2003 and from mid-July to mid-August in 2005 (Table 1).

**Table 1:** Sampling period and general characteristics of brook charr equipped with thermo-sensitive radio transmitters.

Year	*n*	Mean total length (mm)	SD (mm)	Length range (mm)	Sampling period
2003	17	318	23	285–376	19 June –18 July
2005	16	343	28	290–374	19 July –17 August

*n*: number of fish.

### Statistical analyses

#### Spectral decomposition of time series

We used principal coordinates of neighbour matrices (PCNM; [29], [30]) to model temperature time series. The PCNM method, a form of spectral decomposition based on eigenvector extraction, explicitly models spatial or temporal correlation in the data at multiple scales [31]. In this study, we used PCNM to determine which temporal scales are relevant for the patterns of temperature selection in brook charr. This approach was preferred to Fourier analysis or harmonic regression because it allows for missing data (common in our data set) and decomposes spectra into a series of temporal descriptors that can be used as independent variables in a number of statistical methods, such as multiple regression [29]. PCNM analysis generates a series of sinusoidals (hereafter called PCNM) with decreasing periods. Visual inspection of each PCNM variable plotted against time allowed us to roughly define their period. The PCNM are orthogonal (i.e., non-correlated) when a regular sampling grid is used (e.g., one datum per hour). Orthogonality between PCNM is a key property of this approach, facilitating biological interpretation or the formulation of new hypotheses about unknown biological processes revealed by the structure of the data [29], [30]. Temperature data of each individual were modeled with the same set of PCNM (i.e., sinusoidal with exactly the same period), which were constructed using a unique sampling grid for each year, as proposed by Borcard *et al.* [30]. However, because no data were recorded at some time points, our sampling grid was slightly irregular, leading eventually to artificial collinearity among the PCNM [30]. In order to control for this potential problem, we examined the collinearity between independent variables of the model by calculating the variance inflation factor (VIF). A VIF <10 was considered as an indication of low collinearity [32]. PCNM should be used with detrended data if the temporal linear trend is significant [29], [30]. Thus, the effect of time (i.e., the linear trend) was removed from the response variable when necessary. Because of sampling grid irregularities for the two sampling years, PCNM showed some irregularities at the finest temporal scales. Among fine-scale PCNM, only those with unambiguous periodicity were considered in the analyses. Thus, we used the first 180 (smallest period: 8 h) and 360 (smallest period: 4 h) PCNM in 2003 and 2005, respectively. These PCNM were used as independent variables in multiple regressions to model temperature data (dependent variable) for each individual separately. PCNM were selected by forward selection using two stopping criteria: (1) the usual alpha significance level (α = 0.01 in this case) and (2) the adjusted coefficient of determination (R_adj_^2^) computed with all temporal variables [33]. PCNM were built using the “spacemakeR” package [31] in the R statistical language [34].

#### Scalogram – relevant temporal scales

The relative importance of each PCNM for each fish was assessed by its contribution to the R_adj_^2^ of the model. This contribution was expressed by the increase in R_adj_^2^ when a new variable was added to the model by the forward procedure (hereafter called partial R_adj_^2^). Partial R_adj_^2^ values were used to build a scalogram averaged among all individuals within a given year [35]. The scalogram was constructed by plotting the mean (±SD) partial R_adj_^2^ values (Y axis) against each PCNM variable (X axis), ranked by decreasing period. We used this method to highlight the presence of regularities in the periodicities at the population level that helped in defining the relevant temporal scales to understanding brook charr thermoregulatory behaviour.

#### Multivariate analyses – variability within population

Redundancy analyses (RDA) were done on cumulative partial R_adj_^2^ relative to four temporal scales computed for each individual (see Results for definition of the four temporal scales). Sampling year was used as the constraining variable in the RDA. The statistical significance of the relationship between year and the four temporal scales was assessed by means of a permutation test (n = 9999). Data were Hellingher-transformed prior to their use in the RDA [36]. Since we were interested in analyzing the patterns common to both years, we then ran a principal component analysis (PCA) based on the covariance matrix (“vegan” package) on the RDA residuals. This procedure allowed us to (i) define which temporal scales are important at the population level and (ii) eventually highlight the presence of different thermal tactics within the population, controlling for inter-annual variability. Individuals were finally grouped into discrete thermal tactics based on their PCA scores. RDA and PCA analyses were computed with the “vegan” package [37] in the R statistical language.

#### Additional variables

Since the PCNM approach gives information only about the periodicity in the temperature data, we also calculated both the mean temperature and the mean daily thermal amplitude (min–max) for each individual. The mean daily thermal amplitude was calculated for each fish as the difference between the minimum and the maximum temperature for each day averaged for the whole study period. Taken together with PCNM, we used these variables to operationally define the thermal niche experienced by the individuals. The mean daily amplitude and mean temperature were compared among the above-defined thermal tactics and between years by two-way ANOVA.

Radio tracking data were analyzed graphically to illustrate the horizontal component of the diel movements of individuals.

#### Ethics Statement

This study was approved by the Animal Care Committee of the University of Québec at Trois-Rivières (Comité de Bons Soins aux Animaux de l′UQTR - CBSA) (#2002-P.M.7 and # 2005-P.M.15).

## Results

In both study years, Lake Ledoux was stratified during the whole survey period, with the thermocline beginning at depths ranging between two and four meters (Figure S1). The epilimnion was only slightly warmer in 2005 (23.3±1.3°C, mean±SD) than in 2003 (22.7±1.3°C), and thermal profiles were relatively similar between the two years (Figure S1). Tagged fish, whose mean temperatures were 13.3°C (SD 1.8°C) in 2003 and 11.7°C (SD 2.0°C) in 2005, were mainly found in the metalimnion (i.e., the portion of the water column characterized by a temperature gradient >1°C/m; see Figure S1). The mean daily thermal amplitude for the thermographs in 2005 was 0.68±0.31°C (mean±SD), whereas that of fish was 7.56±1.71°C in 2003 and 6.30±1.25°C in 2005.

### Relevant temporal scales

**Figure 1:**
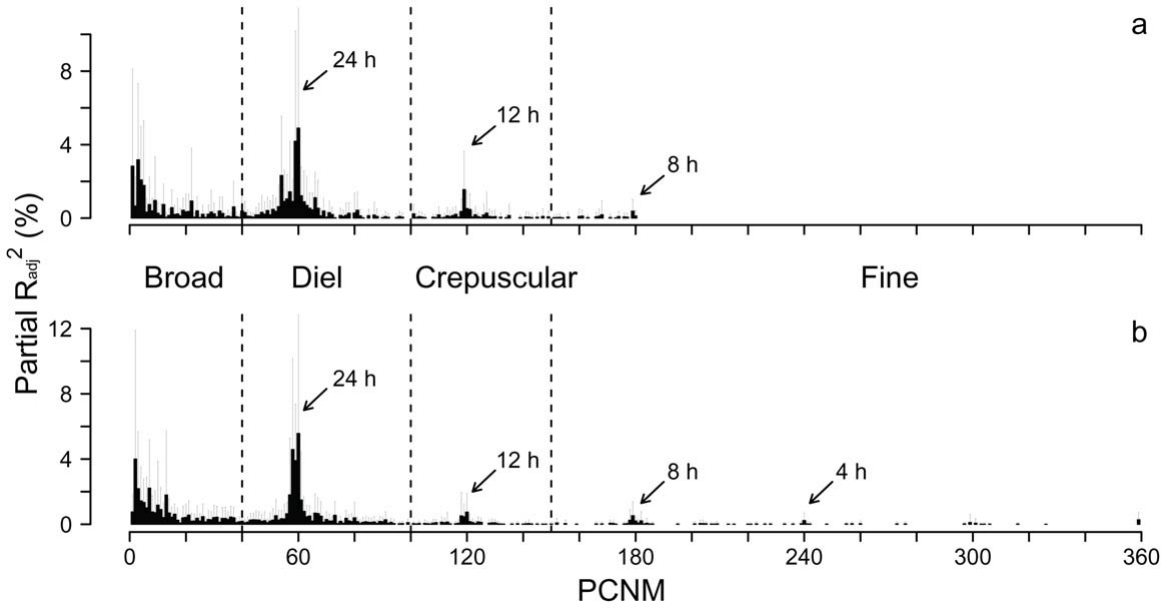
Scalogram based on the average partial R_adj_^2^ distribution of the selected PCNM. Data for fish tracked in 2003 (a) and 2005 (b) (17 individual fish were used in 2003 and 16 in 2005). PCNM periodicity gradually decreases from the left (PCNM 1) to the right (PCNM 360). For each individual fish, PCNM variables were selected by a forward selection procedure (see text). Error bars represent standard deviation of partial R_adj_^2^ among individuals. Arrows indicate approximate PCNM periodicity. Dashed vertical lines are the boundaries of the four temporal scales used in RDA and PCA analyses (see text for details).

The variance inflation factor was always lower than 10 for selected PCNM, confirming weak collinearity among the independent variables. Individual thermal patterns were relatively well fitted by the selected PCNMs, with a mean adjusted coefficient of determination (R_adj_^2^) of 61.4% (SD 15.9%) for the two years combined. It is noteworthy that the periodicities in thermal behaviour identified by the selected PCNM at the population level were qualitatively similar between the two years, as shown by the scalograms (Figure 1). More specifically, the scalograms showed a strong signal around PCNM 60, corresponding to a diel periodicity, and weaker signals at finer temporal scales, with periodicities of 12, 8, and 6 hours (corresponding to PCNM 120, 180, and 240, respectively). The high partial R_adj_^2^ values observed for PCNM 0–10 indicate broadscale thermal patterns, which are more difficult to link to specific patterns because of the span of our sampling period [30]. Based on the shape of the scalograms, we grouped the selected PCNM into four temporal scales; “broad” (periodicity >36 h; PCNM 1–39), “diel” (periodicity 36–14 h; PCNM 40–100), “crepuscular” (periodicity 14–10 h; PCNM 101–150), and “fine” (periodicity <10 h; PCNM 150–180 in 2003, PCNM 150–360 in 2005). These scales roughly correspond to the periodicities defined by Boujard & Leatherland [38] for feeding rhythms in fishes.

### Within-population variability – thermal tactics

**Figure 2:**
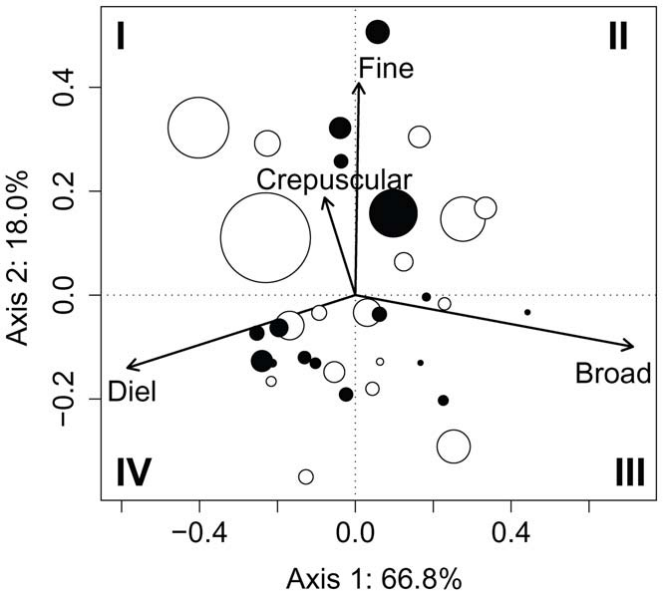
PCA on the RDA residuals (data pooled for the two years) based on the R_adj_^2^ for the PCNMs. Data are pooled for the four temporal scales (i.e., broad, diel, crepuscular, and fine; see text for details). Roman numerals indicate the four quarters of the ordination plot. Open and filled circles refer to individuals in 2003 and 2005, respectively. Circle size is proportional to the mean daily thermal amplitude (see text for details). The values range from 4.5°C to 11.8°C.

RDA analysis was significant (F_1,31_ = 3.56, *P* = 0.03), but sampling year explained only 10.3% of the variation. RDA showed that differences between the two sampling years were mainly due to the crepuscular and fine descriptors (results not shown). In contrast, PCA conducted on RDA residuals revealed clear patterns common to both years (Figure 2). The first axis explained a large portion of the variation (66.8%) and was related to the diel thermal pattern: the more negative the score on the first axis, the more clear-cut the diel cycle was. Individuals showing a diel cycle remained in warmer water during the day and in cooler water during the night (Figures S2, S3, S4, S5, S6). In contrast, those individuals associated with the positive part of the first axis showed a positive correlation with broadscale signals. Therefore, the first axis described a gradient of tactics ranging from individuals with a clear diel pattern (on the left) to individuals with a broad temporal scale pattern (on the right). Although less important than the first axis, the second explained a non-trivial fraction of the variation in the cyclic patterns (18.0%). This axis was correlated with both fine-scale temporal patterns (crepuscular and fine descriptors); individuals with positive scores on this axis exhibited short excursions into warmer water at night or during crepuscular periods (Figures S2, S3, S4, S5, S6).

Since the four temporal scales are strongly correlated to either the first or the second PCA axis, the axes can be used to operationally define four thermal tactics associated with the four quarters of the PCA plot (tactics I–IV; see Figure 2 and Figures S2, S3, S4, S5, S6).

**Figure 3:**
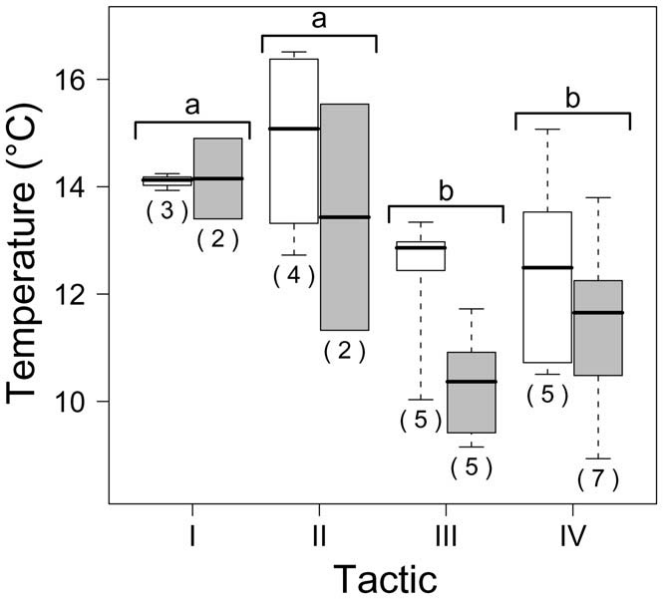
Box plots showing median selected temperature for the two sampling years. Data (*n* = 33) are for the four thermal tactics defined by PCA analysis (I–IV; see Figure 2). The whiskers are extended to extreme values. Sample sizes (individual fish) are indicated below each box plot. Thermal tactics with the same letter are not significantly different (post-hoc Tukey test, *P*<0.05).

Both mean temperature and mean daily thermal amplitude varied among the four tactics described above (Figures 2 and 3). On average, temperatures were slightly lower in 2005 than in 2003 (F_1,28_ = 5.32, *P* = 0.029), but the pattern among thermal tactics I–IV was similar between the two years (no significant year – thermal tactic interaction). More specifically, individuals with positive scores on the second axis (tactics I and II) experienced significantly warmer temperatures than those with negative scores (tactics III and IV; F_3,28_ = 7.58, *P*<0.001; Figure 3). A similar result was found with the mean daily thermal amplitude (F_3,25_ = 7.60, *P*<0.001; results not shown).

Most individuals displaying tactic IV showed a diel cycle characterized by the use of cool waters during the night and warmer waters during the day (Figure S6) and were relatively synchronous in both years (Figure S10). On average, these individuals selected temperatures around 11.8±1.7°C (interannual mean ± SD; Figure S10). The pattern for tactic I individuals was somewhat less clear than for tactic IV, but they shared some similarities, with most individuals in both tactics using warmer waters during the day than during the night. Some exceptions existed within tactic I, with two individuals using cooler waters during the day than at night (Figure S3). In contrast to tactic IV, it was clear that some periodicity appeared around the crepuscular hours in tactic I (Figure S3). For this tactic, the cycles were relatively synchronous in 2005 but not in 2003 (Figure S7). On average, these individuals selected temperatures around 14.6±1.2°C (Figure S7). The periodicity at the crepuscular periods was also evident in tactic II individuals (Figure S4), but no clear diel periodicity was present. These individuals selected temperatures around 14.4±2.1°C on average (Figure S8). Tactic III individuals showed no clear pattern either at the diel or at the crepuscular level (Figure S5). On average, these individuals selected temperatures around 11.3±1.6°C (Figure S9).

### Radio tracking

**Figure 4:**
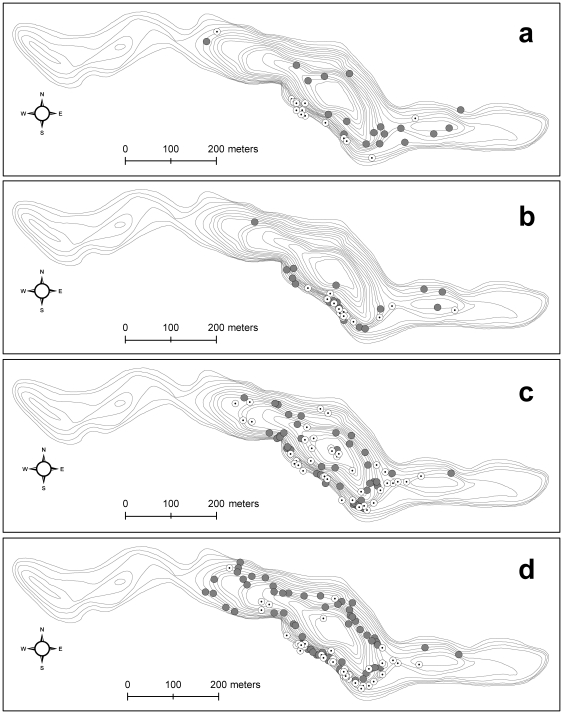
Localizations of individuals from tactics I–IV (a–d) in 2005. Open symbols: day (9 am–3 pm); filled symbols: night (9 pm–3 am). Radio-tracking data are pooled for all individuals that belonged to a given tactic (tactic I: two individuals; tactic II: two individuals; tactic III: five individuals; tactic IV: seven individuals). Isobaths are shown (1 m intervals; maximum depth  = 17 m).

Most radio-tracked individuals (2005 data only) were in the tactic III and IV groups (five and seven, respectively) whereas only a few displayed tactics I and II (two each). Individuals displaying tactic IV aggregated during the day in a section along the south shore and dispersed during the night (Figure 4d), whereas individuals displaying tactic III did not aggregate during the day or night (Figure 4c). These latter individuals were the only ones that preferentially exploited the pelagic zone during the day (Figure 4c). Individuals displaying tactic I aggregated during the day in the same south shore section as tactic IV individuals and then dispersed during the night (Figure 4a). In contrast, results were less clear-cut for tactic II individuals, with most localizations being made in the same section of the south shore during both night and day (Figure 4b).

## Discussion

We found clear differences in mean temperature and thermal cycles among groups of brook charr individuals, suggesting the existence of four tactics within the population. These differences were not merely related to the mean selected temperature since, contrary to our predictions, only half of the population showed a true diel cycle. The observed cycles were clearly not related to daily oscillations in the temperatures of the water column since the daily variation measured by thermographs (Figure S11) was one order of magnitude lower than those experienced by fish (Figures S7, S8, S9, S10). Furthermore, the observed thermal behaviours were consistent between years: the two warm tactics were characterized by both crepuscular and finer periodicities with (I) or without (II) a diel periodicity and the two cool tactics with (IV) or without (III) a diel periodicity. To our knowledge, this is one of the first studies showing the occurrence of different thermal behaviour tactics within a fish population.

Brook charr was mainly found in the metalimnion of our study lake, at temperatures within their specific preferred range [26], suggesting behavioural thermoregulation at the macrohabitat scale for the whole population. However, the warm tactics matched the optimum temperature for growth more closely than the cool tactics (i.e., 14.6°C; [26]), with an average difference of ca. 2°C between the two tactics. This indicates a certain degree of spatial segregation along the vertical axis and is probably a physiologically important difference for a cold-water stenotherm such as brook charr [39]. Since the seminal paper by Beamish [40], both experimental [41], [42] and field [43] studies have shown that different brook trout traits (e.g., respiration rate and growth) are widely affected by temperature changes in the range experienced by fish in our study. These results suggest that the differences in thermal behaviour observed in our study could have important consequences on the fitness of these animals. Both the presence of a crepuscular behaviour and the amplitude of the daily variations in selected temperatures also varied among the tactics. Individuals displaying the warm tactics were crepuscular and made more frequent incursions to suboptimal temperatures (i.e., >20°C, Figures S7, S8, S9, S10) than individuals displaying the cool tactics. The importance of the fine-scale periodicities (the positive part of the second PCA axis; Figure 2) is probably an indication of these incursions into epilimnetic waters. It is possible that such forays occurred in the pelagic zone, but this seems unlikely since this zone is probably less profitable than the littoral zone in terms of food (P. Magnan, unpublish data). Thus, together with their higher average temperature, this trait suggests that these individuals were more likely to make forays to the littoral zone, where temperatures were always >20°C during the study period. However, the thermal costs of these forays probably limit the extent of littoral feeding even for individuals displaying a warm tactic, whose forays into warm-water habitats were usually short. Probably because of this mechanism, individuals made less frequent incursions into shallow waters in 2005, which were warmer than in 2003 (Figure S12) The need to thermoregulate by returning rapidly to optimal temperatures after a feeding foray has been observed in other species [44], [45] and is probably the reason for the relatively short periodicities associated with the warm tactics. The presence of a well-documented trophic polymorphism in Lake Ledoux might be a reasonable explanation behind the presence of warm and cool tactics in this system. However, since the excursions in the shallow (warm) areas of Lake Ledoux (east and west sections; see Figure 4) were only rarely recorded by radio tracking, it is difficult to confirm the differences in the use of the littoral zone among the four tactics. Even though both visual observations and net captures conducted over several years (P. Magnan, pers. obs.) suggest that brook trout commonly use these shallow areas, this was not shown in the radio-tracking data, probably because dawn and dusk periods were not covered in the present study.

Diel vertical migrations (DVM) is often viewed as a way to optimize the energy budget in fishes: individuals feed during the night in relatively warm layers and digest at lower temperatures during the day, thus reducing losses due to basal metabolism [9], [46], [47]. This is opposite to what was found in Lake Ledoux, where most individuals showing a diel cycle spend the day in warmer waters and the night in cooler and deeper waters (i.e., inverse DVM). Even though brook charr is more active during the night than during the day in Lake Ledoux, suggesting nocturnal feeding [27], it displays an inverse DVM in at least 50% of the cases (Figures S6 and S10). It has been shown in laboratory experiments that inverse DVM could be induced in cyprinids by starvation [17], suggesting that food limitation might be a driving force in our oligotrophic system. However, since not all the individuals showed cyclic tactics in Lake Ledoux, it is difficult to explain why this mechanism did not similarly affect all individuals in the population. A sex-related difference in energetic budgets might be a more realistic candidate mechanism since it can explain within-population differences in thermoregulation needs. An example of sexual dimorphism in thermoregulatory behaviour is found in leopard shark (*Triakis semifasciata*), where only females perform inverse DVM [48]. Sex-related differences in thermal behaviour associated with gonad maturation or energy status have also been observed in a variety of organisms, such as grasshoppers [19], lizards [14], and salmonid fish [20]. Since our study was conducted during brook charr gonad maturation, it is reasonable to consider that it affected their thermal behaviour.

Two of the most important patterns, the diel and the crepuscular ones, are related to vertical movements based on the diel cycle and are likely linked to brook charr activity. Previous results from Lake Ledoux have shown that adult brook charr activity is related to diel cycles with peaks at night and during crepuscular periods [27]. Since Bourke et al. [27] used telemetry to measure brook charr activity on the horizontal plane, it is not unlikely that the vertical movements observed in our study might also have a horizontal component (Figure 4): individuals displaying diel tactics (e.g., IV) aggregate during the day and disperse during the night (Figure 4d), in contrast to individuals displaying broadscale tactics (e.g., III), which did not aggregate during the day or night (Figure 4c). The diel horizontal pattern observed for tactic IV is strikingly similar to what was observed by Bourke et al. [27] in Lake Ledoux about one decade before our study: in both studies, individuals aggregated during the day in the same area, which is located close to a steep rocky shore (based on the comparison of Figure 4d with Figures 1, 2, 3 in [27]). Too few individuals displaying tactics I and II were tracked to show differences from tactics III and IV, but these results are in accordance with our operational definition of diel vs. broadscale tactics based on the PCA. Despite these limitations, our results suggest a potential reduction of niche overlap among similarly sized individuals as evidenced by the observed within-population variability in thermoregulatory behaviour.

Because the manipulation of tagged fish must be done rapidly to maximize survival, it was not possible to asses their sex and form through accurate morphological measurements. Therefore, it is not possible to directly relate these factors to the observed tactics. Nevertheless, the interpretation that the tactics observed in our study lake are due to the combined effects of (i) different habitat use by littoral and pelagic individuals and (ii) different energy needs of males and females are reasonable and parsimonious interpretations. Whatever the mechanisms involved, the presence of different tactics within a population was clearly demonstrated and should not be underestimated. Our results suggest that the presence of different tactics contributes to maintaining a spatio-temporal segregation of individuals within the population and, in turn, leads to a better exploitation of the available resources [49]. Since global warming will probably increase thermal habitat segregation in Canadian Shield lakes [50], thermal behaviour will probably evolve as well. A better knowledge of within-population behavioural adaptations to thermal niches might help when elaborating better management strategies by modelling fine-tuned responses of species to global changes [51].

## Supporting Information

Figure S1 Thermal profiles of Lake Ledoux in 2003 (a) and 2005 (b). For each year, only profiles corresponding to the first (solid dots) and last (open dots) days of the survey are shown.
(TIF)

Figure S2 PCA on the RDA residuals (data pooled for the two years) based on the R_adj_^2^ for the PCNMs with fish identified by individual numbers. The numbers refer to the codes for each individual tagged fish in 2003 (numbers in normal font) and 2005 (numbers in bold italic font). The four panels of the PCA were used to define the four behavioural thermoregulatory tactics (I–IV). The same numbers were used to identify individual thermal signatures (Figures S3, S4, S5, S6).
(TIF)

Figure S3 Individual thermal signatures for tactic I. Thermal signatures of individual fish followed from 19 June to 18 July 2003 (numbers in normal font) and from 19 July to 17 August 2005 (numbers in bold italic font). Box plots were used to summarize the temperature data on a daily period (one box plot per hour per fish). The boxplots show median values with the 25^th^ and 75^th^ percentiles. The whiskers show the range of data values that fall within the 1.5 interquartile ranges of either quartile. The circles represent outliers.
(TIF)

Figure S4 Individual thermal signatures for tactic II. Thermal signatures of individual fish followed from 19 June to 18 July 2003 (numbers in normal font) and from 19 July to 17 August 2005 (numbers in bold italic font). Symbols as in Fig. S3.
(TIF)

Figure S5 Individual thermal signatures for tactic III. Thermal signatures of individual fish followed from 19 June to 18 July 2003 (numbers in normal font) and from 19 July to 17 August 2005 (numbers in bold italic font). Symbols as in Fig. S3.
(TIF)

Figure S6 Individual thermal signatures for tactic IV. Thermal signatures of individual fish followed from 19 June to 18 July 2003 (numbers in normal font) and from 19 July to 17 August 2005 (numbers in bold italic font). Symbols as in Fig. S3.
(TIF)

Figure S7 Synchronicity of the thermal signatures for tactic I. a) 2003 (n  =  3 individuals), b) 2005 (n  =  2 individuals). For each tactic (Figures S7, S8, S9, S10), we fitted the pooled individual temperature data using PCNM analyses. We used the *R^2^* statistic to give a rough estimate of the synchronicity in the thermal signatures for each tactic. We expected that a high *R^2^* would be related to a high synchronicity among individuals that belonged to a same tactic (e.g., thermal signals of all the individuals of a given tactic would have the same periodicity and be in phase). Overall, individual temperature data were well fitted by PCNM analyses (*R^2^*  =  61.4±15.9% SD). Thus, we are confident that a poor fit (low *R^2^* value) of the pooled data reflects a lack of synchronicity of the thermal signatures among the individuals rather than a poor individual fit.
(TIF)

Figure S8 Synchronicity of the thermal signatures for tactic II. a) 2003 (n  =  4 individuals), b) 2005 (n  =  2 individuals).
(TIF)

Figure S9 Synchronicity of the thermal signatures for tactic III. a) 2003 (n  =  5 individuals), b) 2005 (n  =  5 individuals).
(TIF)

Figure S10 Synchronicity of the thermal signatures for tactic IV. a) 2003 (n  =  5 individuals), b) 2005 (n  =  7 individuals).
(TIF)

Figure S11 Temporal evolution of the temperature of Lake Ledoux in 2005. The hourly temperature data are shown for the 20 thermographs ranging from 0.5 to 10 m in depth. Grey and black lines represent measurements taken at 1 m intervals starting from 0.5 m and 1 m in depth, respectively. Note that the figure is scaled as in figures S7, S8, S9, S10 to aid comparisons.
(TIF)

Figure S12 Depths selected by individual fish in 2003 (a) and 2005 (b). Individuals were grouped according to their behavioural thermoregulatory tactic (I–IV; see Figure 2). The numbers refer to the codes for each individual tagged fish in 2003 (numbers in normal font) and 2005 (numbers in bold italic font). Depths were interpolated from the thermal profile of the lake (one profile per day) and the temperature data of individuals. The boxplots show median values with 25^th^ and 75^th^ percentiles. The whiskers show the range of data values that fall within the 1.5 interquartile ranges of either quartile. The circles represent outliers.
(TIF)
