# Supplementary figures and images for: Behavioural Thermoregulatory Tactics in Lacustrine Brook Charr, Salvelinus fontinalis
Source: PLoS One. 2011 Apr 7;6(4):e18603. doi: 10.1371/journal.pone.0018603 (PMC3072417; doi:10.1371/journal.pone.0018603)

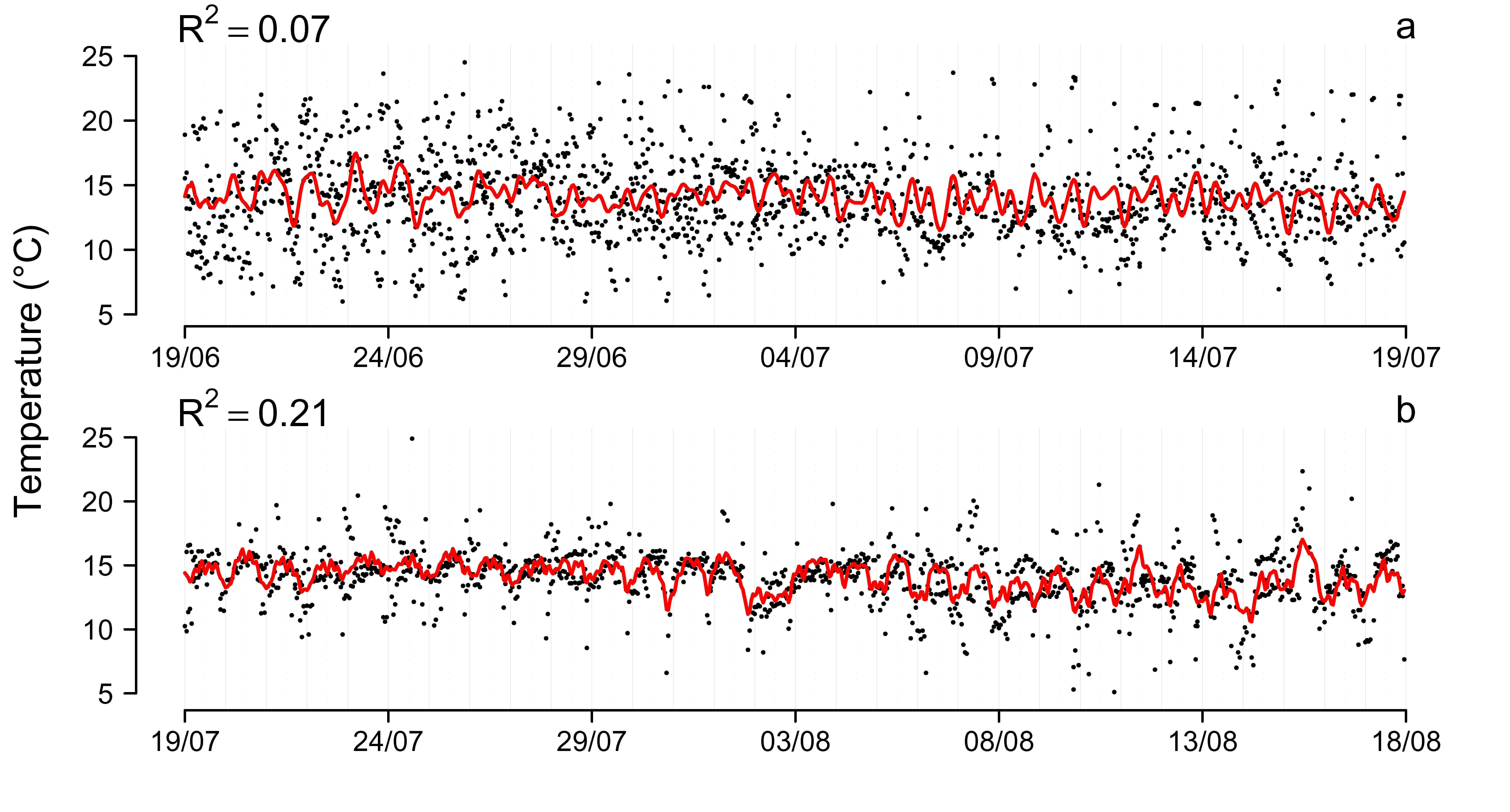

Supplement: Figure S7 — Synchronicity of the thermal signatures for tactic I. a) 2003 (n = 3 individuals), b) 2005 (n = 2 individuals). For each tactic (Figures S7, S8, S9, S10), we fitted the pooled individual temperature data using PCNM analyses. We used the R2 statistic to give a rough estimate of the synchronicity in the thermal signatures for each tactic. We expected that a high R2 would be related to a high synchronicity among individuals that belonged to a same tactic (e.g., thermal signals of all the individuals of a given tactic would have the same periodicity and be in phase). Overall, individual temperature data were well fitted by PCNM analyses (R2 = 61.4±15.9% SD). Thus, we are confident that a poor fit (low R2 value) of the pooled data reflects a lack of synchronicity of the thermal signatures among the individuals rather than a poor individual fit. (TIF) [file pone.0018603.s007.tif]

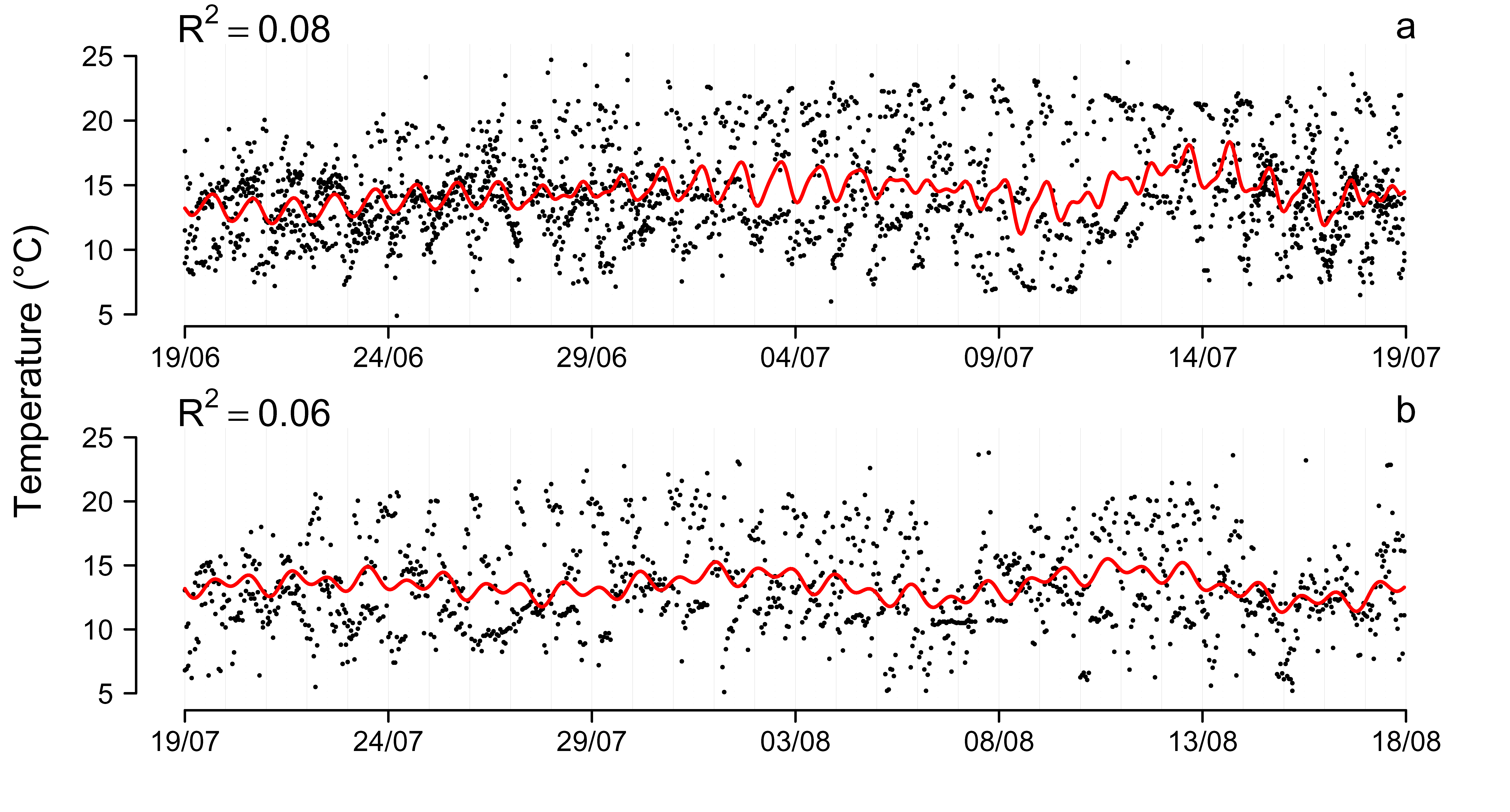

Supplement: Figure S8 — Synchronicity of the thermal signatures for tactic II. a) 2003 (n = 4 individuals), b) 2005 (n = 2 individuals). (TIF) [file pone.0018603.s008.tif]

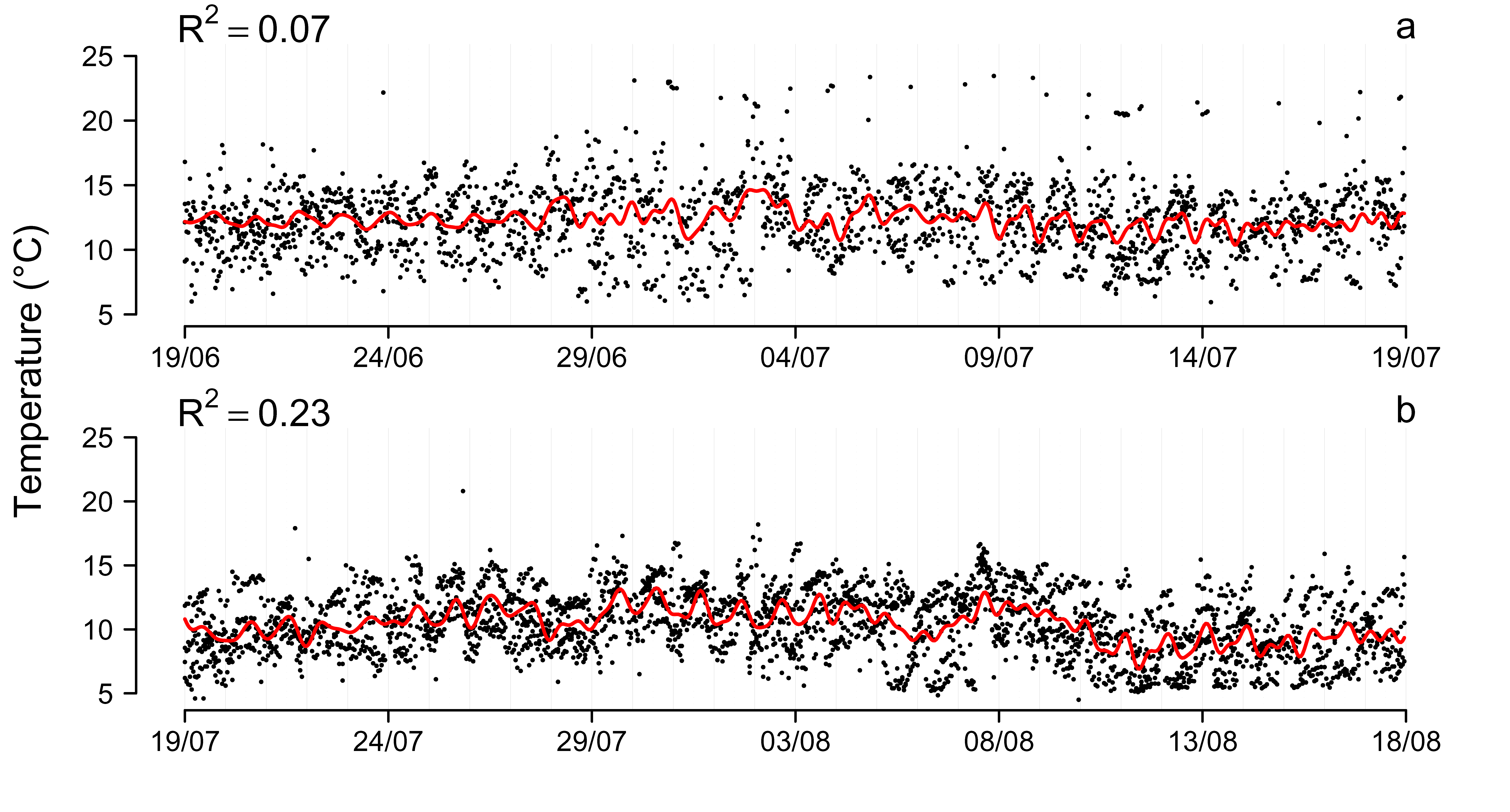

Supplement: Figure S9 — Synchronicity of the thermal signatures for tactic III. a) 2003 (n = 5 individuals), b) 2005 (n = 5 individuals). (TIF) [file pone.0018603.s009.tif]

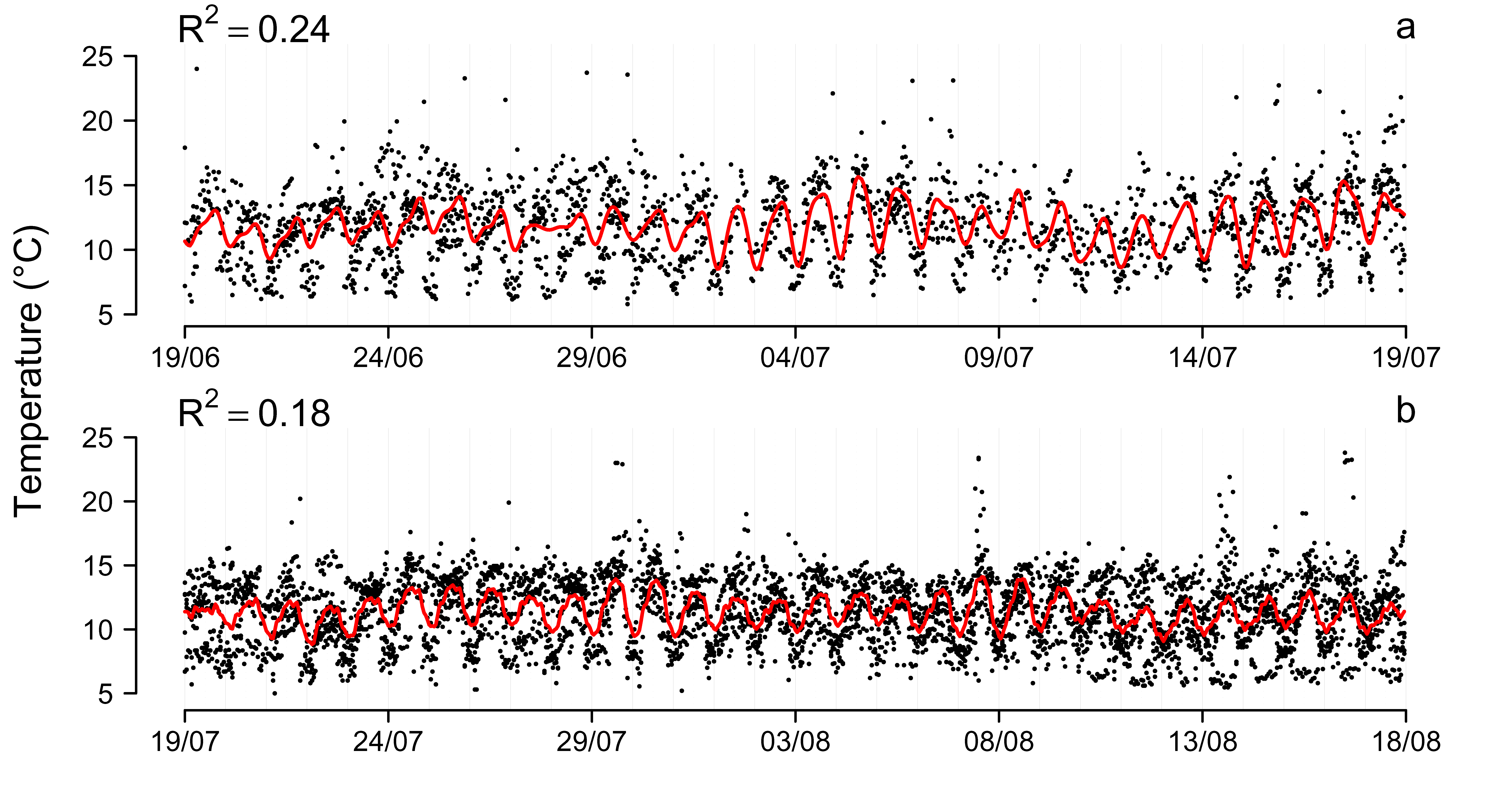

Supplement: Figure S10 — Synchronicity of the thermal signatures for tactic IV. a) 2003 (n = 5 individuals), b) 2005 (n = 7 individuals). (TIF) [file pone.0018603.s010.tif]

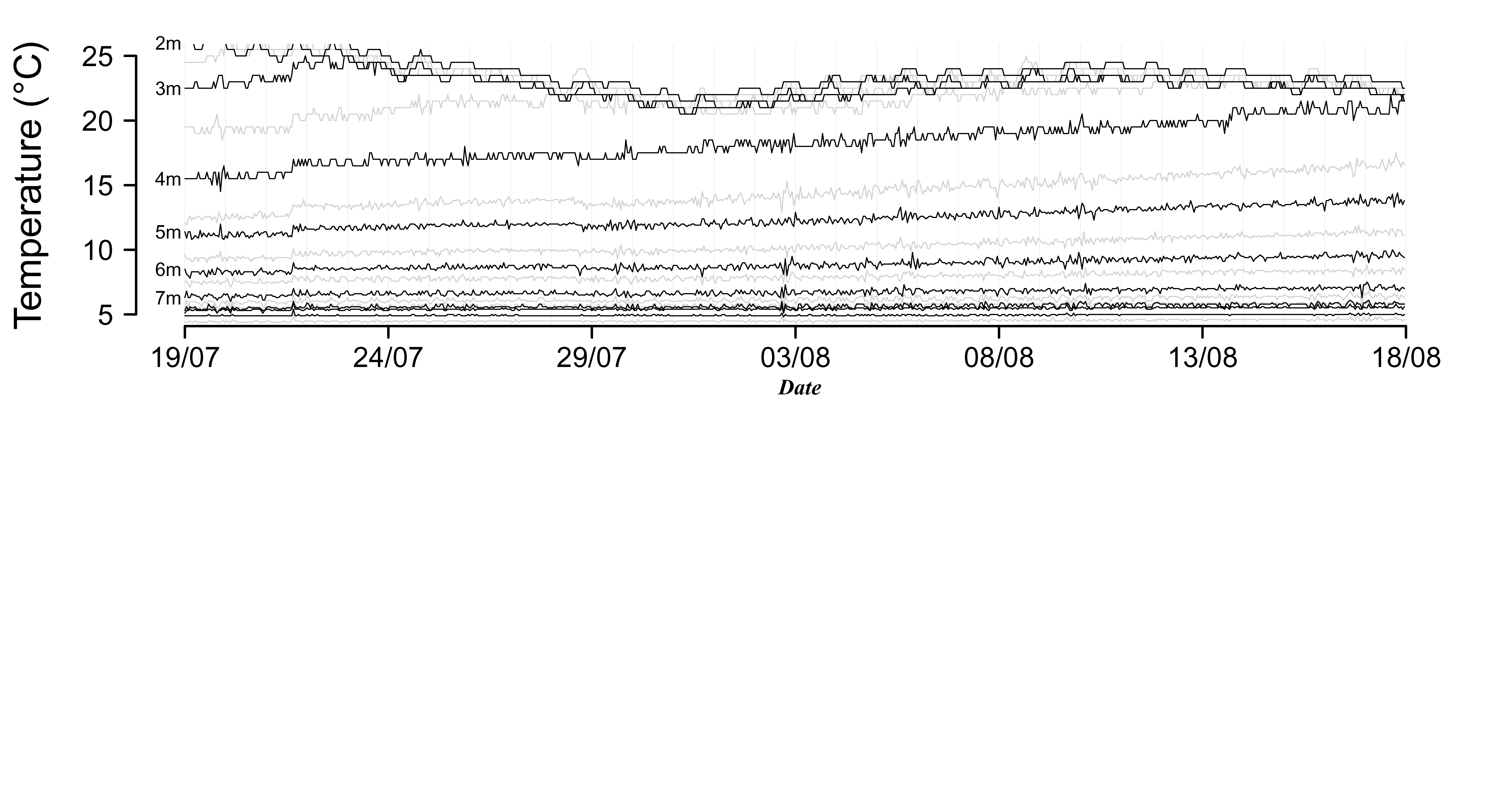

Supplement: Figure S11 — Temporal evolution of the temperature of Lake Ledoux in 2005. The hourly temperature data are shown for the 20 thermographs ranging from 0.5 to 10 m in depth. Grey and black lines represent measurements taken at 1 m intervals starting from 0.5 m and 1 m in depth, respectively. Note that the figure is scaled as in figures S7, S8, S9, S10 to aid comparisons. (TIF) [file pone.0018603.s011.tif]
